# The factors that influence oral health-related quality of life in young adults

**DOI:** 10.1186/s12955-018-1015-7

**Published:** 2018-09-17

**Authors:** Ling Sun, Hai Ming Wong, Colman P. J. McGrath

**Affiliations:** 1 Paediatric Dentistry and Orthodontics, Faculty of Dentistry, The University of Hong Kong, 2/F, Prince Philip Dental Hospital, 34 Hospital Road, Sai Ying Pum, Hong Kong; 2 Periodontology and Public Health, Faculty of Dentistry, The University of Hong Kong, Sai Ying Pum, Hong Kong

**Keywords:** Oral health-related quality of life, Periodontal status, Caries, Malocclusion, Sociodemographic factors, Young adults

## Abstract

**Background:**

Young adulthood is a time when subjects transform their role from a dependent child to an independent social identity. This cross-sectional study aimed to analyze the sociodemographic and clinical factors that may influence the OHRQoL of 18-year-old young adults.

**Methods:**

A representative sample was selected from Hong Kong. Periodontal status and caries were examined according to WHO criteria. Four orthodontic indices were used to assess malocclusion. The oral health impact profile (OHIP-14) was used to measure OHRQoL. Adjusted OR was calculated by ordinal logistic regression.

**Results:**

A total of 300 eligible subjects (165 females, 135 males) were recruited. Females had more severe caries than males; however, gender was not a significant factor of OHRQoL. Household income affected OHRQoL more than parents’ education did: household income had effects on physical pain, psychological discomfort, psychological disability, and the total OHIP; while parents’ education had some effects on functional limitation, physical pain and psychological discomfort. As for clinical factors, unhealthy periodontal conditions were more prevalent than caries (94.67% vs. 59.00%); however, both of them showed no effect on OHRQoL. Malocclusion had a negative effect on OHRQoL; the most affected subscales were psychological discomfort and psychological disability.

**Conclusion:**

In this study, family ecosocial factors and malocclusion had an effect on OHRQoL. Among the family ecosocial factors, it was household income that had the most effect on OHRQoL. Malocclusion mainly affected the subscales of psychological discomfort and psychological disability. Gender, periodontal status and caries had no effect on young adults’ OHRQoL.

## Background

The concept of health-related quality of life emerged in the late 1960s, while the concept of oral health-related quality of life (OHRQoL) appeared in the early 1980s [1]. OHRQoL measures not only oral symptoms and functional limitations, but also their impacts on patients’ psychosocial status. Psychosocial status is liable to change according to age; hence quality of life is a “dynamic construct” that is likely to change overtime [2].

During adolescence, subjects’ physical and psychological statuses develop rapidly. Subjects become more aware of their appearance; their emotion is vulnerable and changeable [3]. In adulthood, subjects’ perceptions of inside and outside world are relatively stable [4]. Young adulthood is a time that bridges adolescence and adulthood, in which subjects transform their role from a dependent child to an independent social identity [5]. To date, only a few studies have been conducted on the factors that influence young adults’ OHRQoL; a final conclusion has not been reached in this area [6–11].

This article is a cross-sectional study aimed to analyze the sociodemographic and clinical factors that may influence young adults’ OHRQoL. The sample of this study was randomly selected from 18-year-old students in Hong Kong.

## Methods

### Measurement instruments

The short form of oral health impact profile (OHIP-14) was used to assess OHRQoL. The following 7 dimensions are measured: functional limitation, physical pain, psychological discomfort, physical disability, psychological disability, social disability, and handicap. For each question subjects are asked how frequently they had experienced the impact in the preceding 12 months. The responses followed a Likert-type scale coded as ‘never’ = 0; ‘hardly ever’ = 1; ‘occasionally’ = 2; ‘fairly often’ = 3; and ‘very often’ = 4. The total score can be calculated as the sum of the item scores, generating scores from 0 to 56 for OHIP-14, with higher scores indicating worse OHRQoL [12].

Community Periodontal Index (CPI) and the Decayed, Missing and Filled Teeth (DMFT) were used to measure periodontal and caries conditions according to the criteria of WHO [13]. Significant Caries Index (SiC index) was also used to classify caries. The one third of the population with the highest caries score is selected and the mean DMFT for this subgroup constitutes the SiC Index value [14].

Index of Orthodontic Treatment Need (IOTN), Dental Aesthetic Index (DAI), Index of Complexity, Outcome and Need (ICON), and Peer Assessment Rating (PAR) were used to assess orthodontic treatment need and complexity [15–20].

IOTN includes Dental health component (DHC) and Aesthetic component (AC). DHC has 5 grades (no need to very great need) and the worst occlusal trait is recorded to allocate the grade. AC is comprised of 10 front view photographs, which represents 10 scales of dental attractiveness. The IOTN (DHC) or IOTN (AC) grading can be further categorized into three orthodontic treatment groups (DHC 1–2 or AC 1–4, no need; DHC 3 or AC 5–7, borderline need; DHC 4–5 or AC 8–10, definite need) [21, 22].

The index of DAI is calculated by multiplying the measurements of 10 occlusal traits by their weights; the addition of their products and the addition of a constant number, 13, is the final DAI score. It can be categorized into 4 scales of orthodontic severity and treatment need (≤ 25, normal or minor malocclusion-no treatment need or slight need; 26–30, definite malocclusion-treatment selective; 31–35: severe malocclusion-treatment highly desirable; ≥ 36: very severe (handicapping) malocclusion-treatment mandatory) [18].

ICON is used to evaluate treatment need, treatment outcome and complexity [19]. Its aesthetic score is assessed using IOTN (AC). Five occlusal trait scores are multiplied by their respective weights and summed to calculate the ICON score. The ICON score can be scaled into 2 categories for treatment need (≤ 43 No; > 43 Yes), and 5 categories for orthodontic complexity (< 29 easy; 29–50, mild; 51–63 moderate; 64–77 difficult; > 77 very difficult).

PAR is an estimate of how far a case deviates from normal. The concept is to assign a score to 11 components of occlusal traits that make up a malocclusion. The individual scores are summed together to obtain an overall total, representing the degree a case deviates from normal occlusion. Generally a measure of 10 or less indicates an acceptable alignment and occlusion, and 5 or less suggests an almost ideal occlusion [17].

### Study population and data collection

Cluster randomized trial was used in this study. The sampling frame was all local secondary schools in Hong Kong (by law all children are required to attend secondary school). A random sample of 45 schools (approximately 10% of all local secondary schools) was selected from 18 districts in Hong Kong, SAR. Students born between April 1st and May 31st, 1997 were invited to participate in the oral health survey conducted by Faculty of Dentistry, the University of Hong Kong. The sample of this study was selected from the birth cohort of “children of 1997” [23].

This study was part of a longitudinal study that was planned to follow subjects from age 12 to 18. Sample size was calculated based on a previous study [24–26]. The prevalence of orthodontic treatment need (ICON) was 80.3%; the mean CPQ scores (SD) were respectively 20.1 (14.0) and 14.8 (15.0) for “with treatment need” group and “without treatment need” group; α = 0.05, and 1-β = 0.8. With a lost rate of 30% at each follow-up and the design effect for cluster sampling considered, the sample sizes at ages 12, 15, and 18 should be 237, 166, and 116, respectively.

It should be noted that at age 18, not only subjects who were followed up from age 12 came to the survey again, but also some new subjects, who did not show up in the past surveys, were willing to participate in this 18-year-old survey. Therefore, this article is a cross-sectional analysis of all these 18-year-old subjects; the longitudinal analysis for the subjects who were followed up from age 12 to 18 was demonstrated in another article.

Students’ oral health status was examined using an intra-oral disposable mouth mirror with a built-in LED light source. The same trained and calibrated examiner performed the oral examination according to the criteria of WHO [13]. Front-view dental photos were taken by extracting lips using oral retractors to assess IOTN (AC). Dental impressions were collected and the plaster models were sent to OrthoLab (Poland) to make digital models. Software O3DM (version3.8.5 (c) by OrthoLab, Poland) was used to analyse digital models by the same examiner. Reassessments were performed among 10% randomly selected samples after 2 weeks of first assessment to test intra-examiner’s reliability.

Systematic health information, dental treatment history, ecosocial factors including father’s education, mother’s education, and household income were collected from a self-completed questionnaire. OHRQoL was assessed by inviting participants to answer questions in OHIP-14. Subjects were excluded from the final analysis if they were systemically unhealthy, had orthodontic treatment history, or had oral diseases other than caries, periodontitis and malocclusion.

### Ethics, consent and permissions

The ethical approval of this study was granted by the Institutional Review Board of the University of Hong Kong/Hospital Authority Hong Kong West Cluster (UW 09–453). A written consent from parents/primary caregivers and a verbal consent from students were obtained from all participants.

### Statistical methods

Intra-examiner reliability was tested by kappa values for CPI, weighted kappa for IOTN (DHC) and IOTN (AC), and Intra-class correlation coefficient (ICC) for DMFT, DAI score, and ICON score.

Missing data only existed in some questions of family information. The problem was addressed as follows: the missing data were first checked in the data of age 15, and then in age 12; if the data were available at age 15 or age 12, they were used in this study. As such, only 6 subjects had missing data of one or two questions, which were filled with the mode of the corresponding data at age 18.

Mann-Whitney U test was used to analyze whether there was a difference of oral health status between females and males; independent samples t test was used to detect the difference of mean DMFT between females and males.

The effects of sociodemographic and clinical factors on OHRQoL were analyzed with parameters set as follows:

1. Dependent variables: for bivariate analysis, dependent variables were set as the subscale and the total scores of OHIP-14. For ordinal regression, if the data could be separated into four groups, the cut-offs were set as quartiles; if not, the cut-offs were set as medians; higher ranks represented poorer quality of life.
2. Independent variables: gender, father’s education level (primary school graduate or below, secondary school, post-secondary or above), mother’s education level (levels set as father’s education), household income (total monthly income below HK$10000, HK$10001-HK$20000, HK$20001-HK$30000, HK$30001-HK$40000, HK$40001 or above), periodontal status, caries experience, and orthodontic treatment need.
3. Bivariate analysis: comparison between two samples used the Mann-Whitney U test, others used the Kruskal-Wallis H test.
4. Multivariate analysis: ordinal logistic regression was used to calculate adjusted odds ratios (OR). To avoid interaction effect, orthodontic treatment needs measured by different orthodontic indices were entered into regression separately.

## Results

There were 383 subjects participating in the 18-year-old survey; of these, 300 (165 females, 135 males) were eligible for the final analysis. A total of 83 subjects were excluded from this study, of whom 33 were systematically unhealthy, and 50 were with orthodontic history or without oral impressions. Of the eligible subjects in this study, 204 (114 females and 90 males) participated in both 15-year-old and 18-year-old surveys; 188 (106 females and 82 males) participated in all three surveys.

Kappa value for CPI was 0.789; weighted kappa for IOTN (DHC) and IOTN (AC) were 0.918 and 0.790; ICC for DAI score, ICON score and DMFT were 0.821, 0.820 and 0.996.

The oral health status of subjects is presented in Table 1. In this 18-year-old sample, the mean DMFT (SD) was 1.92 (2.373) and the SiC index value (SD) was 4.72 (2.021). Unhealthy periodontal conditions were more prevalent than caries (94.67% vs. 59.00%). The prevalence of orthodontic treatment need was 46.33% measured by IOTN (DHC), 19.67% by IOTN (AC), 57.00% by DAI, 34.33% by ICON, and 46.00% by PAR. There was no difference of oral health status between females and males, except for caries. The prevalence of caries was not different between females and males; but the situation of caries was more severe in females than in males (*p* = 0.036 and 0.003 for SiC index and DMFT, respectively).

**Table 1:** Profile of 18-year-old participants

	Female		Male		Total		P
	N	Percentage	N	Percentage	N	Percentage	
IOTN (DHC) treatment need							
No need	89	53.94%	72	53.33%	161	53.67%	0.876
Borderline need	35	21.21%	28	20.74%	63	21.00%	
Definite need	41	24.85%	35	25.93%	76	25.33%	
IOTN (AC) treatment need							
No need	132	80.00%	109	80.74%	241	80.33%	0.952
Borderline need	23	13.94%	15	11.11%	38	12.67%	
Definite need	10	6.06%	11	8.15%	21	7.00%	
DAI severity and treatment need							
Normal or minor malocclusion-no treatment need or slight need	75	45.45%	54	40.00%	129	43.00%	0.238
Definite malocclusion-treatment selective	50	30.30%	42	31.11%	92	30.67%	
Severe malocclusion-treatment highly desirable	25	15.15%	20	14.81%	45	15.00%	
Very severe (handicapping) malocclusion-treatment mandatory	15	9.09%	19	14.07%	34	11.33%	
ICON treatment need							
No	108	65.45%	89	65.93%	197	65.67%	0.932
Yes	57	34.55%	46	34.07%	103	34.33%	
ICON complexity							
Easy	47	28.48%	42	31.11%	89	29.67%	0.858
Mild	84	50.91%	63	46.67%	147	49.00%	
Moderate	14	8.48%	15	11.11%	29	9.67%	
Difficult	14	8.48%	8	5.93%	22	7.33%	
Very difficult	6	3.64%	7	5.19%	13	4.33%	
PAR							
Almost ideal occlusion	41	24.85%	30	22.22%	71	23.67%	0.833
Acceptable occlusion	48	29.09%	43	31.85%	91	30.33%	
Malocclusion	76	46.06%	62	45.93%	138	46.00%	
Periodontal status							
CPI score = 0	10	6.06%	6	4.44%	16	5.33%	0.536
CPI score > 0	155	93.94%	129	95.56%	284	94.67%	
CPI score < 2	14	8.48%	7	5.19%	21	7.00%	0.266
CPI score > =2	151	91.52%	128	94.81%	279	93.00%	
Caries experience							
< SiC Index value	135	81.82%	122	90.37%	257	85.67%	0.036*
> =SiC Index value	30	18.18%	13	9.63%	43	14.33%	
DMFT = 0	61	36.97%	62	45.93%	123	41.00%	0.117
DMFT> 0	104	63.03%	73	54.07%	177	59.00%	
DMFT		Mean (SD)		Mean (SD)		Mean (SD)	
	165	2.28 (2.603)	135	1.48 (1.977)	300	1.92 (2.373)	0.003**

P: comparison for DMFT used the independent samples t test; others used the Mann-Whitney U test

SiC Index: Significant Caries Index; SiC index value (SD) was 4.72 (2.021)

The results of bivariate analysis are presented in Table 2. No difference of OHRQoL existed between females and males. The total OHIP score showed a gradient descent across the rates of both parents’ education and household income; however, significant results only existed in household income. As for the subscales of OHIP-14, father’s education showed effects on functional limitation and psychological discomfort; mother’s education showed effects on physical pain; household income showed the most significant effects, which were presented on physical pain, psychological discomfort, and psychological disability.

**Table 2:** Bivariate analysis between the factors and the OHIP-14

	N	Functional limitation			Physical pain			Psychological discomfort			Physical disability			Psychological disability			Social disability			Handicap			OHIP total score		
		Mean (SD)	Median (IQR)	P	Mean (SD)	Median (IQR)	P	Mean (SD)	Median (IQR)	P	Mean (SD)	Median (IQR)	P	Mean (SD)	Median (IQR)	P	Mean (SD)	Median (IQR)	P	Mean (SD)	Median (IQR)	P	Mean (SD)	Median (IQR)	P
Gender																									
F	165	0.40 (0.875)	0.00 (0)	0.222	1.16 (1.181)	1.00 (2)	0.979	1.09 (1.383)	0.00 (2)	0.692	0.44 (0.879)	0.00 (1)	0.612	0.84 (1.256)	0.00 (1)	0.880	0.28 (0.731)	0.00 (0)	0.574	0.28 (0.721)	0.00 (0)	0.663	4.49 (5.176)	3.00 (5)	0.872
M	135	0.47 (0.845)	0.00 (1)		1.15 (1.149)	1.00 (2)		0.90 (1.036)	1.00 (2)		0.45 (0.808)	0.00 (1)		0.70 (0.947)	0.00 (1)		0.29 (0.645)	0.00 (0)		0.26 (0.634)	0.00 (0)		4.22 (4.270)	3.00 (5)	
Total	300	0.43 (0.861)	0.00 (0)		1.16 (1.165)	1.00 (2)		1.01 (1.240)	1.00 (2)		0.44 (0.846)	0.00 (1)		0.78 (1.127)	0.00 (1)		0.29 (0.692)	0.00 (0)		0.27 (0.682)	0.00 (0)		4.37 (4.784)	3.00 (5)	
Father’s education																									
Primary school graduate or below	48	0.77 (1.115)	0.00 (2)	0.008**	1.40 (1.180)	1.00 (2)	0.207	1.38 (1.282)	1.00 (2)	0.031*	0.63 (1.104)	0.00 (1)	0.264	0.75 (0.978)	0.00 (1)	0.061	0.29 (0.798)	0.00 (0)	0.952	0.23 (0.592)	0.00 (0)	0.978	5.44 (5.539)	3.50 (7)	0.159
Secondary school graduate or below	208	0.34 (0.770)	0.00 (0)		1.12 (1.140)	1.00 (2)		0.96 (1.231)	0.00 (2)		0.37 (0.703)	0.00 (1)		0.85 (1.184)	0.00 (2)		0.28 (0.668)	0.00 (0)		0.28 (0.723)	0.00 (0)		4.20 (4.533)	3.00 (5)	
College graduate or above	44	0.48 (0.876)	0.00 (1)		1.09 (1.254)	1.00 (2)		0.82 (1.187)	0.00 (1)		0.61 (1.083)	0.00 (1)		0.45 (0.951)	0.00 (1)		0.30 (0.701)	0.00 (0)		0.25 (0.576)	0.00 (0)		4.00 (5.012)	2.00 (5)	
Mother’s education																									
Primary school graduate or below	45	0.60 (0.939)	0.00 (1)	0.193	1.38 (1.230)	1.00 (2)	0.005**	1.09 (1.311)	1.00 (2)	0.540	0.33 (0.674)	0.00 (1)	0.370	0.76 (1.004)	0.00 (1)	0.323	0.22 (0.670)	0.00 (0)	0.376	0.13 (0.505)	0.00 (0)	0.114	4.51 (4.655)	3.00 (5)	0.079
Secondary school graduate or below	224	0.41 (0.863)	0.00 (0)		1.19 (1.165)	1.00 (2)		1.02 (1.247)	1.00 (2)		0.49 (0.903)	0.00 (1)		0.83 (1.191)	0.00 (1)		0.32 (0.717)	0.00 (0)		0.31 (0.728)	0.00 (0)		4.57 (4.943)	3.00 (6)	
College graduate or above	31	0.35 (0.709)	0.00 (0)		0.58 (0.886)	0.00 (1)		0.77 (1.087)	0.00 (2)		0.26 (0.575)	0.00 (0)		0.45 (0.723)	0.00 (1)		0.16 (0.523)	0.00 (0)		0.16 (0.523)	0.00 (0)		2.74 (3.406)	2.00 (4)	
Household income																									
below HK$10,000	24	0.38 (0.824)	0.00 (0)	0.475	1.54 (1.351)	1.00 (2)	0.005**	1.29 (1.334)	1.00 (3)	0.011*	0.29 (0.690)	0.00 (0)	0.661	0.83 (1.129)	0.00 (2)	0.003**	0.33 (0.761)	0.00 (0)	0.660	0.21 (0.509)	0.00 (0)	0.190	4.88 (4.911)	3.00 (7)	0.004**
HK$10,001-HK$20,000	170	0.47 (0.905)	0.00 (1)		1.22 (1.175)	1.00 (2)		1.14 (1.316)	1.00 (2)		0.49 (0.885)	0.00 (1)		0.89 (1.214)	0.00 (1)		0.32 (0.757)	0.00 (0)		0.31 (0.730)	0.00 (0)		4.84 (5.120)	3.00 (6)	
HK$20,001-HK$30,000	49	0.51 (0.916)	0.00 (1)		1.20 (1.099)	1.00 (2)		0.98 (1.164)	1.00 (2)		0.41 (0.674)	0.00 (1)		0.92 (1.152)	0.00 (2)		0.24 (0.596)	0.00 (0)		0.31 (0.822)	0.00 (0)		4.57 (4.500)	3.00 (6)	
HK$30,001-HK$40,000	26	0.31 (0.788)	0.00 (0)		1.04 (1.113)	1.00 (2)		0.50 (0.906)	0.00 (1)		0.42 (0.987)	0.00 (0)		0.46 (0.761)	0.00 (1)		0.35 (0.689)	0.00 (0)		0.31 (0.549)	0.00 (1)		3.38 (4.177)	2.00 (6)	
Over HK$40,001	31	0.23 (0.560)	0.00 (0)		0.52 (0.890)	0.00 (1)		0.52 (0.851)	0.00 (1)		0.35 (0.877)	0.00 (0)		0.16 (0.454)	0.00 (0)		0.10 (0.301)	0.00 (0)		0.03 (0.180)	0.00 (0)		1.90 (2.508)	1.00 (3)	
Periodontal status																									
CPI score = 0	16	0.25 (0.577)	0.00 (0)	0.515	0.94 (1.181)	0.50 (2)	0.374	0.38 (0.500)	0.00 (1)	0.059	0.44 (0.629)	0.00 (1)	0.522	0.69 (0.946)	0.00 (2)	0.836	0.19 (0.544)	0.00 (0)	0.573	0.25 (0.577)	0.00 (0)	0.886	3.13 (3.612)	3.00 (6)	0.249
CPI score > 0	284	0.44 (0.874)	0.00 (0)		1.17 (1.165)	1.00 (2)		1.04 (1.260)	1.00 (2)		0.44 (0.858)	0.00 (1)		0.78 (1.138)	0.00 (1)		0.29 (0.700)	0.00 (0)		0.27 (0.688)	0.00 (0)		4.44 (4.837)	3.00 (5)	
CPI score < 2	21	0.29 (0.644)	0.00 (0)	0.515	0.95 (1.203)	0.00 (2)	0.319	0.52 (0.680)	0.00 (1)	0.123	0.33 (0.577)	0.00 (1)	0.904	0.71 (0.902)	0.00 (2)	0.955	0.24 (0.539)	0.00 (0)	0.953	0.24 (0.539)	0.00 (0)	0.864	3.29 (3.608)	3.00 (6)	0.284
CPI score > =2	279	0.44 (0.875)	0.00 (0)		1.17 (1.163)	1.00 (2)		1.04 (1.266)	1.00 (2)		0.45 (0.863)	0.00 (1)		0.78 (1.144)	0.00 (1)		0.29 (0.703)	0.00 (0)		0.27 (0.692)	0.00 (0)		4.45 (4.856)	3.00 (5)	
Caries experience																									
DMFT = 0	123	0.50 (0.909)	0.00 (1)	0.247	1.05 (1.193)	1.00 (2)	0.095	1.02 (1.248)	1.00 (2)	0.871	0.52 (0.961)	0.00 (1)	0.399	0.79 (1.216)	0.00 (1)	0.707	0.37 (0.793)	0.00 (0)	0.138	0.30 (0.689)	0.00 (0)	0.341	4.54 (5.231)	2.00 (6)	0.659
DMFT> 0	177	0.38 (0.825)	0.00 (0)		1.23 (1.142)	1.00 (2)		1.00 (1.239)	0.00 (2)		0.39 (0.754)	0.00 (1)		0.77 (1.065)	0.00 (1)		0.23 (0.608)	0.00 (0)		0.25 (0.679)	0.00 (0)		4.25 (4.458)	3.00 (5)	
< SiC Index value	257	0.46 (0.901)	0.00 (1)	0.386	1.16 (1.149)	1.00 (2)	0.828	1.02 (1.253)	1.00 (2)	0.601	0.47 (0.888)	0.00 (1)	0.317	0.80 (1.157)	0.00 (1)	0.439	0.31 (0.720)	0.00 (0)	0.244	0.28 (0.667)	0.00 (0)	0.170	4.50 (4.948)	3.00 (5)	0.500
> =SiC Index value	43	0.26 (0.539)	0.00 (0)		1.16 (1.271)	1.00 (2)		0.91 (1.171)	0.00 (2)		0.26 (0.492)	0.00 (0)		0.63 (0.926)	0.00 (1)		0.16 (0.485)	0.00 (0)		0.21 (0.773)	0.00 (0)		3.58 (3.594)	3.00 (4)	
IOTN (DHC) treatment need																									
No need	161	0.35 (0.769)	0.00 (0)	0.213	1.07 (1.152)	1.00 (2)	0.189	0.82 (1.156)	0.00 (2)	0.005**	0.46 (0.851)	0.00 (1)	0.722	0.60 (0.964)	0.00 (1)	0.018*	0.27 (0.661)	0.00 (0)	0.971	0.25 (0.635)	0.00 (0)	0.709	3.82 (4.441)	2.00 (5)	0.073
Borderline need	63	0.51 (0.931)	0.00 (1)		1.37 (1.222)	2.00 (2)		1.37 (1.286)	1.00 (2)		0.44 (0.963)	0.00 (0)		0.87 (1.100)	0.00 (2)		0.30 (0.775)	0.00 (0)		0.21 (0.600)	0.00 (0)		5.06 (5.127)	3.00 (7)	
Definite need	76	0.54 (0.972)	0.00 (1)		1.17 (1.136)	1.00 (2)		1.11 (1.312)	1.00 (2)		0.41 (0.734)	0.00 (1)		1.08 (1.383)	1.00 (2)		0.30 (0.693)	0.00 (0)		0.36 (0.828)	0.00 (0)		4.96 (5.100)	3.00 (6)	
IOTN (AC) treatment need																									
No need	241	0.39 (0.815)	0.00 (0)	0.250	1.18 (1.161)	1.00 (2)	0.149	0.93 (1.185)	0.00 (2)	0.004**	0.43 (0.829)	0.00 (1)	0.828	0.71 (1.086)	0.00 (1)	0.011*	0.27 (0.636)	0.00 (0)	0.407	0.23 (0.595)	0.00 (0)	0.586	4.15 (4.535)	3.00 (5)	0.039*
Borderline need	38	0.55 (1.058)	0.00 (1)		1.26 (1.245)	1.00 (2)		1.58 (1.348)	1.50 (2)		0.47 (0.951)	0.00 (1)		1.21 (1.234)	1.00 (2)		0.42 (0.858)	0.00 (0)		0.47 (1.033)	0.00 (0)		5.97 (5.450)	4.00 (6)	
Definite need	21	0.67 (0.966)	0.00 (2)		0.71 (1.007)	0.00 (1)		0.81 (1.436)	0.00 (2)		0.52 (0.873)	0.00 (1)		0.71 (1.271)	0.00 (1)		0.29 (0.956)	0.00 (0)		0.33 (0.796)	0.00 (0)		4.05 (5.895)	2.00 (5)	
DAI																									
Normal or minor malocclusion	129	0.29 (0.687)	0.00 (0)	0.0499*	1.15 (1.200)	1.00 (2)	0.487	0.81 (1.097)	0.00 (1)	0.024*	0.39 (0.721)	0.00 (1)	0.511	0.60 (0.955)	0.00 (1)	0.0500	0.26 (0.641)	0.00 (0)	0.363	0.23 (0.644)	0.00 (0)	0.136	3.72 (4.176)	3.00 (4)	0.051
Definite malocclusion	92	0.52 (0.955)	0.00 (1)		1.08 (1.131)	1.00 (2)		1.02 (1.284)	0.50 (2)		0.47 (0.988)	0.00 (0)		0.79 (1.163)	0.00 (1)		0.26 (0.693)	0.00 (0)		0.21 (0.565)	0.00 (0)		4.35 (5.000)	2.00 (6)	
Severe and very severe (handicapping) malocclusion	79	0.56 (0.971)	0.00 (1)		1.27 (1.151)	1.00 (2)		1.32 (1.354)	1.00 (2)		0.51 (0.860)	0.00 (1)		1.04 (1.295)	1.00 (2)		0.37 (0.771)	0.00 (0)		0.41 (0.840)	0.00 (0)		5.46 (5.296)	4.00 (7)	
ICON treatment need																									
No	197	0.36 (0.760)	0.00 (0)	0.100	1.08 (1.129)	1.00 (2)	0.111	0.80 (1.132)	0.00 (1)	0.000**	0.42 (0.827)	0.00 (1)	0.572	0.60 (0.998)	0.00 (1)	0.000**	0.24 (0.616)	0.00 (0)	0.297	0.21 (0.602)	0.00 (0)	0.061	3.72 (4.279)	2.00 (4)	0.001**
Yes	103	0.57 (1.016)	0.00 (1)		1.31 (1.221)	1.00 (2)		1.40 (1.346)	1.00 (2)		0.49 (0.884)	0.00 (1)		1.11 (1.283)	1.00 (2)		0.37 (0.816)	0.00 (0)		0.38 (0.806)	0.00 (0)		5.62 (5.431)	4.00 (6)	
PAR																									
Almost ideal or Acceptable occlusion	162	0.33 (0.738)	0.00 (0)	0.035*	1.01 (1.089)	1.00 (2)	0.019*	0.77 (1.076)	0.00 (1)	0.001**	0.35 (0.734)	0.00 (0)	0.060	0.58 (0.883)	0.00 (1)	0.006**	0.20 (0.533)	0.00 (0)	0.058	0.20 (0.577)	0.00 (0)	0.068	3.43 (3.843)	2.00 (5)	0.000**
Malocclusion	138	0.55 (0.975)	0.00 (1)		1.33 (1.228)	1.00 (2)		1.28 (1.362)	1.00 (2)		0.56 (0.952)	0.00 (1)		1.01 (1.326)	0.50 (2)		0.39 (0.832)	0.00 (0)		0.36 (0.781)	0.00 (0)		5.48 (5.504)	4.00 (7)	

P: *p* value of nonparametric tests; *: *p* < 0.05, **p < 0.01

Nonparametric tests: comparison between two samples used the Mann-Whitney U test; others used the Kruskal-Wallis H test

SiC Index: Significant Caries Index; SiC index value (SD) was 4.72 (2.021)

In all subscales of OHIP-14, subjects with unhealthy periodontal conditions had a higher score than those with healthy conditions. However, statistical results showed that periodontal status, as well as caries, had no effect on any subscale of OHIP-14. When malocclusion was classified into two groups by ICON and PAR, in all subscales of OHIP-14, subjects with malocclusion had a higher score than those without malocclusion. Nevertheless, not all subscales presented a significant result. The subscales of psychological discomfort and psychological disability were the most detected subscales: all indices showed a significant result in these two subscales. Among the used indices, PAR detected the most significant results.

The results of ordinal regression were almost the same with bivariate analysis (Table 3). Gender showed no effect on any subscale of OHIP-14. As for family ecosocial factors, household income showed the most effect on OHRQoL. The affected subscales were physical pain, psychological discomfort, psychological disability, and the total OHIP score. In these subscales, higher household incomes were associated with lower likelihoods of having worse OHRQoL; however, statistical results showed that only the last one or two groups with the highest incomes had significant effects on OHRQoL. Take the subscale of psychological discomfort for example. When compared with the first group, the last two groups had lower likelihoods of having problems in this subscale (adjusted OR = 0.25 and 0.26, respectively). Parents’ education showed less effect on OHRQoL than household income did. Both father’s and mother’s education showed some positive effects, with father’s education on functional limitation and psychological discomfort, and mother’s education on physical pain.

**Table 3:** Ordinal regression of associations between the factors and OHIP-14

	Functional limitation		Physical pain		Psychological discomfort		Physical disability		Psychological disability		Social disability		Handicap		OHIP total score	
	Adjusted OR (95%CI)	P	Adjusted OR (95%CI)	P	Adjusted OR (95%CI)	P	Adjusted OR (95%CI)	P	Adjusted OR (95%CI)	P	Adjusted OR (95%CI)	P	Adjusted OR (95%CI)	P	Adjusted OR (95%CI)	P
Sociodemographic status																
Gender																
F^a^																
M	1.41 (0.81, 2.48)	0.228	1.01 (0.66, 1.56)	0.950	0.93 (0.60, 1.45)	0.753	1.09 (0.64, 1.86)	0.740	1.12 (0.69, 1.83)	0.646	1.23 (0.66, 2.29)	0.507	1.16 (0.61, 2.20)	0.649	0.94 (0.61, 1.43)	0.757
Father’s education																
Primary school graduate or below^a^																
Secondary school graduate or below	0.43 (0.20, 0.94)	0.034*	0.64 (0.34, 1.20)	0.161	0.49 (0.25, 0.93)	0.031*	0.57 (0.26, 1.23)	0.152	0.89 (0.44, 1.82)	0.755	1.03 (0.40, 2.65)	0.946	0.71 (0.27, 1.82)	0.470	0.66 (0.35, 1.25)	0.206
College graduate or above	1.04 (0.33, 3.23)	0.946	1.56 (0.61, 4.00)	0.354	0.54 (0.21, 1.43)	0.218	1.34 (0.44, 4.08)	0.604	0.51 (0.17, 1.52)	0.225	1.39 (0.36, 5.44)	0.633	1.52 (0.40, 5.81)	0.540	0.78 (0.31, 1.97)	0.599
Mother’s education																
Primary school graduate or below^a^																
Secondary school graduate or below	0.62 (0.27, 1.41)	0.258	1.02 (0.53, 1.95)	0.960	1.51 (0.76, 2.99)	0.238	1.47 (0.63, 3.44)	0.375	1.08 (0.52, 2.26)	0.832	1.40 (0.52, 3.77)	0.505	3.21 (0.98, 10.51)	0.054	1.33 (0.69, 2.56)	0.387
College graduate or above	0.55 (0.14, 2.16)	0.391	0.32 (0.11, 0.96)	0.043*	1.71 (0.56, 5.18)	0.345	0.65 (0.16, 2.63)	0.551	1.71 (0.50, 5.87)	0.391	0.60 (0.11, 3.39)	0.566	1.23 (0.19, 7.93)	0.824	0.98 (0.34, 2.81)	0.974
Household income																
below HK$10,000^a^																
HK$10,001-HK$20,000	1.57 (0.52, 4.80)	0.427	0.71 (0.32, 1.58)	0.405	0.76 (0.34, 1.70)	0.502	1.53 (0.52, 4.49)	0.441	1.09 (0.45, 2.65)	0.851	0.70 (0.23, 2.13)	0.527	0.90 (0.27, 3.00)	0.865	0.86 (0.39, 1.90)	0.712
HK$20,001-HK$30,000	2.34 (0.67, 8.20)	0.183	0.78 (0.31, 1.94)	0.593	0.71 (0.28, 1.80)	0.467	1.56 (0.46, 5.25)	0.472	1.16 (0.42, 3.25)	0.772	0.60 (0.16, 2.24)	0.449	0.60 (0.15, 2.47)	0.482	0.91 (0.37, 2.27)	0.844
HK$30,001-HK$40,000	0.87 (0.19, 4.05)	0.858	0.61 (0.22, 1.74)	0.360	0.25 (0.08, 0.81)	0.020*	1.11 (0.27, 4.52)	0.880	0.55 (0.17, 1.83)	0.329	1.01 (0.24, 4.22)	0.984	1.49 (0.34, 6.51)	0.595	0.52 (0.18, 1.47)	0.218
Over HK$40,001	0.67 (0.14, 3.15)	0.615	0.19 (0.06, 0.58)	0.004**	0.26 (0.08, 0.81)	0.021*	0.74 (0.17, 3.20)	0.692	0.20 (0.05, 0.82)	0.026*	0.30 (0.06, 1.66)	0.169	0.10 (0.01, 1.05)	0.055	0.22 (0.07, 0.67)	0.008**
Periodontal and caries status																
Periodontal status																
CPI score = 0^a^																
CPI score > 0	1.58 (0.40, 6.14)	0.512	1.96 (0.74, 5.21)	0.176	2.73 (0.90, 8.29)	0.076	0.70 (0.23, 2.09)	0.519	1.44 (0.49, 4.25)	0.508	1.56 (0.33, 7.40)	0.574	1.07 (0.28, 4.13)	0.918	1.72 (0.67, 4.45)	0.261
CPI score < 2^a^																
CPI score > =2	1.43 (0.44, 4.64)	0.552	1.97 (0.84, 4.62)	0.118	2.20 (0.87, 5.52)	0.095	1.06 (0.38, 2.94)	0.917	1.19 (0.47, 3.02)	0.711	0.92 (0.29, 2.96)	0.887	1.01 (0.31, 3.28)	0.981	1.77 (0.77, 4.05)	0.178
Caries experience																
DMFT = 0^a^																
DMFT> 0	0.78 (0.44, 1.38)	0.392	1.41 (0.91, 2.19)	0.125	1.01 (0.64, 1.59)	0.963	0.90 (0.53, 1.54)	0.704	1.13 (0.69, 1.86)	0.629	0.64 (0.35, 1.19)	0.160	0.73 (0.39, 1.38)	0.330	0.99 (0.64, 1.52)	0.956
< SiC Index value^a^																
> =SiC Index value	0.90 (0.39, 2.07)	0.808	0.96 (0.52, 1.75)	0.882	0.90 (0.48, 1.69)	0.735	0.88 (0.40, 1.93)	0.746	0.70 (0.35, 1.41)	0.320	0.60 (0.22, 1.65)	0.326	0.47 (0.16, 1.43)	0.185	0.88 (0.48, 1.61)	0.681
Malocclusion																
IOTN (DHC) treatment need																
No need^a^																
Borderline need	1.24 (0.61, 2.52)	0.549	1.41 (0.82, 2.42)	0.218	2.22 (1.28, 3.85)	0.005**	0.60 (0.30, 1.22)	0.159	1.52 (0.82, 2.79)	0.181	0.93 (0.42, 2.05)	0.858	0.73 (0.31, 1.71)	0.469	1.49 (0.87, 2.54)	0.147
Definite need	1.59 (0.83, 3.07)	0.164	1.10 (0.66, 1.83)	0.720	1.45 (0.86, 2.46)	0.163	0.82 (0.44, 1.54)	0.538	1.81 (1.01, 3.24)	0.046*	1.04 (0.50, 2.16)	0.922	1.04 (0.49, 2.19)	0.927	1.43 (0.87, 2.37)	0.162
IOTN (AC) treatment need																
No need^a^																
Borderline need	1.35 (0.60, 3.06)	0.468	0.97 (0.52, 1.82)	0.921	2.44 (1.29, 4.61)	0.006**	0.93 (0.42, 2.05)	0.852	2.62 (1.25, 5.49)	0.011*	1.46 (0.63, 3.38)	0.383	1.28 (0.53, 3.08)	0.580	2.04 (1.08, 3.84)	0.027*
Definite need	1.61 (0.59, 4.36)	0.352	0.36 (0.14, 0.89)	0.026*	0.55 (0.22, 1.39)	0.204	1.18 (0.44, 3.18)	0.747	0.76 (0.28, 2.06)	0.589	0.51 (0.11, 2.37)	0.394	1.48 (0.44, 4.99)	0.527	0.83 (0.36, 1.92)	0.665
DAI severity and treatment need																
Normal or minor malocclusion^a^																
Definite malocclusion	1.69 (0.86, 3.31)	0.127	0.94 (0.57, 1.55)	0.804	1.36 (0.81, 2.29)	0.247	0.86 (0.45, 1.62)	0.637	1.56 (0.88, 2.76)	0.128	0.74 (0.35, 1.59)	0.445	0.95 (0.43, 2.10)	0.906	1.15 (0.70, 1.89)	0.576
Severe malocclusion and very severe (handicapping) malocclusion	1.89 (0.96, 3.75)	0.067	1.14 (0.68, 1.93)	0.623	2.02 (1.18, 3.46)	0.011*	1.21 (0.64, 2.27)	0.559	1.77 (0.98, 3.22)	0.060	1.39 (0.68, 2.86)	0.371	1.82 (0.86, 3.85)	0.117	1.79 (1.06, 3.01)	0.029*
ICON treatment need																
No^a^																
Yes	1.42 (0.81, 2.51)	0.224	1.35 (0.86, 2.10)	0.189	2.49 (1.58, 3.94)	0.000**	1.08 (0.63, 1.87)	0.779	2.48 (1.48, 4.16)	0.001**	1.29 (0.69, 2.42)	0.422	1.77 (0.93, 3.37)	0.082	2.08 (1.33, 3.24)	0.001**
PAR score range																
Almost ideal or Acceptable occlusion^a^																
Malocclusion	1.62 (0.93, 2.84)	0.091	1.47 (0.96, 2.25)	0.079	2.09 (1.34, 3.26)	0.001**	1.40 (0.82, 2.37)	0.214	1.70 (1.04, 2.77)	0.033*	1.55 (0.84, 2.87)	0.165	1.56 (0.83, 2.95)	0.170	1.94 (1.26, 2.96)	0.002**

Statistical method: Ordinal regression (link function: logit; model: main effects), each orthodontic index adopted one separate ordinal regression; dependent variable: Physical pain, Psychological discomfort, OHIP scores (1: scores ≤ first quartile; 2: first quartile < scores ≤ second quartile; 3: second quartile < scores ≤ third quartile; 4: scores > third quartile); other domain scores (1: scores ≤ median; 2: scores > median)

a: reference group; OR: odds ratio; CI: confidence interval; *: *P* < 0.05. **: *P* < 0.01

N: sample size; adjusted OR: malocclusions adjusted for gender, father’s education level (primary school graduate or below, secondary school, post-secondary or above), mother’s education level (levels set as father’s education), household income (below HK$10000, HK$10001-HK$20000, HK$20001-HK$30000, HK$30001-HK$40000, HK$40001 or above), caries experience (DMFT = 0, DMFT >0), and periodontal status (CPI score = 0, CPI score > 0); gender, socioeconomic status, periodontal and caries status adjusted for the previous variables and malocclusion measured by PAR deviation (almost ideal occlusion, acceptable occlusion, malocclusion). SiC Index: Significant Caries Index; SiC index value (SD) was 4.72 (2.021)

Periodontal status and caries did not show any effect on subjects’ OHRQoL after adjusting the effects of other factors. As for malocclusion, when classified into 2 groups by PAR or ICON, subjects with malocclusion had a higher likelihood of having a worse experience in all subscales of OHIP-14. Nevertheless, significant results were almost only shown in the subscales of psychological discomfort and psychological disability. It was also noticed that only the severe levels of malocclusion had an effect on OHRQoL. For example, when malocclusion was classified by DAI, there was no difference between the “definite malocclusion” group and the “normal or minor malocclusion” group, but the “severe and very severe malocclusion” group was associated with 1.79 times the likelihood of having a worse experience in the total OHIP when compared with the “normal or minor malocclusion” group (*p* = 0.029, Table 3).

## Discussion

This study is part of a longitudinal study that followed subjects from age 12 to 18. When comparing the results of this study with those at age 12 and age 15, sociodemographic factors showed an inconsistent effect on OHRQoL [27, 28]. First, gender was an influence factor of OHRQoL at both ages 12 and 15; while it was no longer an influence factor at age 18. Second, among family ecosocial factors, mother’s education had the most effect on children’s OHRQoL at age 12; whereas it was household income that had the most effect at age 18, although it had little effect at ages 12 and 15. Third, father’s education had a negative effect on children’s OHRQoL at age 12 but a positive effect at age 18. These results suggest that subjects of different age could have different experiences on the same condition, which support the hypothesis that quality of life is a “dynamic construct” that is likely to change overtime [2].

Gender showed no effect on OHRQoL in this study. Similarly, four studies from India, Korea, Saudi Arabia and Malaysia respectively included subjects aged between 18 to 22, 18 to 32, 21 to 25, and 15 to 25 years; their results also showed that gender had little effect on OHRQoL [8–11]. Among family factors, household income presented the most effect on subjects’ OHRQoL. A study assessed socioeconomic inequalities on OHRQoL in 8765 adults aged 21 years and over in England, Wales and Northern Ireland; the result also showed that probabilities of poor oral health and bad oral impacts were significantly higher for participants in lower income quintiles than those in the highest income level [29].

Unhealthy periodontal conditions were more prevalent than caries in all three surveys; and the prevalence of unhealthy periodontal conditions was increased slightly across the three surveys. However, the influence of periodontal status on OHRQoL was inconsistent as well: at age 12 and 15, periodontal status showed an effect on OHRQoL; while at age 18, it showed no effect on OHRQoL. A recent study from Brazil also reported an inconsistent impact of periodontal conditions on OHRQoL: the presence of bleeding had an impact on the domains of EWB and SWB of CPQ_11–14_ in 286 schoolchildren at age 12 [30]; however, when 170 children were followed from age 12 to 15, the presence of bleeding had no impact on CPQ scores [31].

At age 12, no difference of caries was found between females and males; at age 15, the prevalence of caries was higher in females than in males; while at age 18, females had more severe caries than males did. Nevertheless, caries showed little effect on OHRQoL in all three surveys. This result may not be applied to other geographical regions. For example, a study from Japan investigated a group of university students and reported that caries was directly associated with OHRQoL [6]. Hong Kong has mature preventive and treatment conditions for caries, where untreated caries is not very prevalent; hence the symptoms of caries do not harm subjects’ OHRQoL significantly. This result also indicates that although DMFT and SiC index are the common indices for caries assessment, other indices that could differentiate active caries from well-treated caries should be more helpful to determine the effect of caries on OHRQoL. For example, some studies recommended that one of such indices is International Caries Detection and Assessment System (ICDAS), which could discriminate between incipient lesions and lesions encompassing larger portions of the tooth [32, 33].

The effect of malocclusion on OHRQoL was detected across the three surveys of this study. Our systematic review showed that up to February 18, 2016, there were 13 studies testing the impact of untreated malocclusion on OHIP scores [34]. No matter which orthodontic indices were used, most of them reported that untreated malocclusion had an impact on OHIP scores; only 2 studies showed negative results [35, 36]. All subscales of OHIP questionnaire could be affected and the most reported subscale was psychological discomfort. This result was supported by this study: malocclusion could affect OHIP-14 scores; the most detected subscales were psychological discomfort and psychological disability.

Physical pain, psychological discomfort and psychological disability were the most affected subscales in this study; whereas handicap was a subscale that was not affected at all. In OHIP-14, the subscales of physical pain, psychological discomfort and disability demonstrate subjects may have painful aching and uncomfortable eating; they may be self-conscious, unsatisfied, or embarrassed because of their teeth. While the subscale of handicap demonstrates subjects may be unable to function or work because of their teeth. Therefore, the influence factors like household income and malocclusion could affect subjects’ physical and psychological status. However, these effects were not profound enough to hazard their daily lives.

Although the population-based sample used in this study waived the bias of the convenient samples selected from dental clinics, the disadvantages of this study cannot be overlooked. First, this is only a cross-sectional analysis; further longitudinal analysis of this research should provide more helpful evidence. Second, this study was based on a sample selected from Hong Kong. Our systematic review showed that different regions could present different results of OHRQoL [34]. Therefore, when comparing this study with other studies, the differences of national, geographical, cultural, and economical situations need to be considered.

## Conclusion

The influence factors of OHRQoL were studied in a representative sample of 18-year-old subjects. Females had more severe caries than males did; however, gender was not a significant influence factor of OHRQoL. Among family ecosocial factors, household income affected subjects’ OHRQoL most, while parents’ education only had some positive effects on subscales of functional limitation, physical pain and psychological discomfort. Caries and unhealthy periodontal conditions had no effect on OHRQoL; while malocclusion had a negative effect on OHRQoL. The influence factors mainly affected the subscales of physical pain, psychological discomfort and psychological disability.

## Abbreviations

AC: Aesthetic component
CPI: Community Periodontal Index
DAI: Dental aesthetic index
DHC: Dental health component
DMFT: Decayed, Missing and Filled Teeth
ICON: Index of complexity, outcome and need
IOTN: Index of orthodontic treatment need
OHIP: Oral health impact profile
OHRQoL: Oral health-related quality of life
OR: Odds ratio
PAR: Peer assessment rating
SD: Standard deviation
SE: Standard error
SiC index: Significant caries index
WHO: World Health Organization
